# The relationship between cortical network structure and the corresponding state space dynamics

**DOI:** 10.1186/1471-2202-12-S1-P345

**Published:** 2011-07-18

**Authors:** Nicole Voges, Laurent Perrinet

**Affiliations:** 1 INCM, UMR6193 CNRS, Aix-Marseille Université, Marseille, France; 2 current address: Funktionelle Sehforschung/Elektrophysiologie, Universitäts-Augenklinik, Freiburg, Germany

## 

Most studies on the dynamics of recurrent cortical networks are either based on purely random wiring or neighborhood couplings [1-3]. Neuronal wiring in the cortex, however, shows a complex spatial pattern composed of local and long-range patchy connections, i.e., spatially clustered synapses [4,5].

We ask to what extent such geometric traits influence the 'idle' dynamics of cortical network models. Assuming an enlarged spatial scale we consider distinct network architectures, ranging from purely random or purely locally coupled neurons to distance dependent connectivities that also include patchy projections. Approximately 50000 neurons are spatially embedded in a 2D sheet of cortex with a side length of five millimeters in order to account for remotely established synapses. Neurons are implemented as conductance based integrate-and-fire neurons with distance-dependent synaptic delays. Network dynamics are simulated with NEST/PyNN [6].

Analyzing the characteristic measures describing spiking neuronal networks (e.g. the correlation coefficient or the coefficient of variation), we explore and compare the phase spaces and activity patterns of our simulation results. As expected from random networks, different dynamical states (e.g. synchronous regular 'SR' or asynchronous irregular 'AI' firing) occur in dependence of the input rate and the relation between exc. and inh. synaptic strengths [1-3]. Non-random networks, however, exhibit higher firing rates, sharper transitions, as well as various types of complex network activities. For example, the amount of local connections clearly influences the boundaries at which the network switches from high (SR) to low (AI) activity. Distance-dependent connectivity structures induce 'new' raster plots, e.g., oblique stripes or spiral structures representing planar and spherical wave propagation. To better describe such activity patterns we computed a delay-dependent correlation coefficient. Such spike patterns indicate a spatio-temporal spread of activity that random networks cannot account for. Furthermore, to determine stability and signal propagation properties, we applied spatially restricted activity injections. Depending on the network architecture, the dynamics may change from AI to SR or wave-like activity, and then switch back or not.

We conclude that (the amount of) local distance-dependent connections is an important structural feature of cortical networks since it induces rather complex activity patterns compared to random connectivities. However, we found no clear differences in the dynamics of networks with randomly distributed compared to spatially clustered long-range projections. Further analysis is needed to explore the functional aspects of patchy projection patterns.
